# Recent advances in the synthesis of hierarchically mesoporous TiO_2_ materials for energy and environmental applications

**DOI:** 10.1093/nsr/nwaa021

**Published:** 2020-02-14

**Authors:** Wei Zhang, Yong Tian, Haili He, Li Xu, Wei Li, Dongyuan Zhao

**Affiliations:** Department of Chemistry, State Key Laboratory of Molecular Engineering of Polymers, Shanghai Key Laboratory of Molecular Catalysis and Innovative Materials, Laboratory of Advanced Materials, and iChEM, Fudan University, Shanghai 200433, China; Department of Chemistry, State Key Laboratory of Molecular Engineering of Polymers, Shanghai Key Laboratory of Molecular Catalysis and Innovative Materials, Laboratory of Advanced Materials, and iChEM, Fudan University, Shanghai 200433, China; Department of Chemistry, State Key Laboratory of Molecular Engineering of Polymers, Shanghai Key Laboratory of Molecular Catalysis and Innovative Materials, Laboratory of Advanced Materials, and iChEM, Fudan University, Shanghai 200433, China; Department of Chemistry, State Key Laboratory of Molecular Engineering of Polymers, Shanghai Key Laboratory of Molecular Catalysis and Innovative Materials, Laboratory of Advanced Materials, and iChEM, Fudan University, Shanghai 200433, China; Department of Chemistry, State Key Laboratory of Molecular Engineering of Polymers, Shanghai Key Laboratory of Molecular Catalysis and Innovative Materials, Laboratory of Advanced Materials, and iChEM, Fudan University, Shanghai 200433, China; Department of Chemistry, State Key Laboratory of Molecular Engineering of Polymers, Shanghai Key Laboratory of Molecular Catalysis and Innovative Materials, Laboratory of Advanced Materials, and iChEM, Fudan University, Shanghai 200433, China

**Keywords:** hierarchically mesoporous, TiO_2_, energy, environment

## Abstract

Because of their low cost, natural abundance, environmental benignity, plentiful polymorphs, good chemical stability and excellent optical properties, TiO_2_ materials are of great importance in the areas of physics, chemistry and material science. Much effort has been devoted to the synthesis of TiO_2_ nanomaterials for various applications. Among them, mesoporous TiO_2_ materials, especially with hierarchically porous structures, show great potential owing to their extraordinarily high surface areas, large pore volumes, tunable pore structures and morphologies, and nanoscale effects. This review aims to provide an overview of the synthesis and applications of hierarchically mesoporous TiO_2_ materials. In the first section, the general synthetic strategies for hierarchically mesoporous TiO_2_ materials are reviewed. After that, we summarize the architectures of hierarchically mesoporous TiO_2_ materials, including nanofibers, nanosheets, microparticles, films, spheres, core-shell and multi-level structures. At the same time, the corresponding mechanisms and the key factors for the controllable synthesis are highlighted. Following this, the applications of hierarchically mesoporous TiO_2_ materials in terms of energy storage and environmental protection, including photocatalytic degradation of pollutants, photocatalytic fuel generation, photoelectrochemical water splitting, catalyst support, lithium-ion batteries and sodium-ion batteries, are discussed. Finally, we outline the challenges and future directions of research and development in this area.

## INTRODUCTION

Since the first discovery of photocatalytic water splitting on a TiO_2_ electrode under ultraviolet (UV) light, TiO_2_ materials have been widely investigated over the past few decades due to their unique properties such as non-toxicity, abundance, easy availability and stability [1,2]. For the moment, TiO_2_ materials present great potential in applications from the conventional areas (e.g. pigment, cosmetic and toothpaste) to the latest developed areas including catalysis, energy storage and conversion, biomedicine, environmental remediation and so on [3,4]. Beyond all question, TiO_2_ materials offer new candidates to overcome the energy, environmental and health challenges facing humanity today.

Not only the intrinsic electronic structures but also the micro-/nano-structures of TiO_2_ materials affect their physical and chemical properties [5–7]. Various TiO_2_ nanomaterials with different structures have been fabricated and applied in different areas and reveal excellent performances. Among them, mesoporous TiO_2_ materials, especially with hierarchically mesoporous structures, have received increasing interest due to their attractive features, such as high surface areas, large pore volumes, tunable pore structures and nano-confined effects [8–11]. Those features enable the high performances of hierarchically mesoporous TiO_2_ materials in many areas. The high surface area can provide abundant active sites for surface- or interface-related processes such as adsorption and catalysis. The large pore volume has shown great potential in the loading of guest species and the accommodation of structural change, and the porous structure can facilitate the diffusion of reactants and products, which is of benefit for reaction kinetics [12–14]. In the past, many comprehensive reviews have summarized the synthesis, properties and applications of TiO_2_-based nanomaterials, but an overview of the architectural diversity of hierarchically mesoporous TiO_2_ materials and the structure–performance relationship is lacking. Here, we try to focus on those points overlooked by previous reviews. First, the generally synthetic routes for hierarchically mesoporous TiO_2_ materials are reviewed briefly. After that, the critical issues for the controllable synthesis of hierarchically mesoporous TiO_2_ materials with different geometries, including nanofibers, nanosheets, spheres, microparticles, films, core–shell and multi-level structures, are summarized. In the third section, applications of hierarchically mesoporous TiO_2_ materials in energy- and environment-related areas, and the structure–performance relationship, are discussed. Finally, we present a brief conclusion and some perspectives on the future development of this area.

## SYNTHETIC STRATEGIES

Generally, the synthetic methods for hierarchically mesoporous TiO_2_ materials can be classified into four categories: template-free, soft-template, hard-template and multiple-template routes [15,16]. The template-free route is facile and the mesopore voids stem from the aggregation of nanoscale building blocks, but it usually produces products with randomly distributed mesopores. The soft-template method is based on the self-assembly between amphiphilic surfactant molecules and TiO_2_ precursors. In this approach, the interaction between TiO_2_ precursors and surfactant molecules is crucial to the formation of mesostructures. To create a hierarchically porous structure, dual templates or post-treatment, such as calcination or ultrasonication, is necessary. The advantage of the soft-template strategy is that the resultant materials possess some attractive features, including controllable mesostructures and pore sizes, tunable morphologies, and easy processing. However, this method is highly sensitive to the reaction condition and the products suffer from the low crystallinity. The hard-template approach, also known as nanocasting, uses preformed nanostructures as templates, such as mesoporous materials, photonic crystals and biotemplates. The most significant merit of this route is that the resultant materials are highly crystalline. Nevertheless, this approach is tedious and time-consuming. The multiple-template is a general and facile method to produce hierarchically (macro/meso, meso/meso, meso/micro) porous materials. Different synthetic approaches can produce hierarchically mesoporous TiO_2_ materials with different geometries (Table 1), resulting in architectural diversity.

**Table 1.** A comparison of synthetic methods for hierarchically mesoporous TiO_2_ materials.

Methods	Advantages	Disadvantages	Applied architectures
Template-free method	Simple; Easily processable; Highly crystalline products	Products with disordered structures	Nanofibers; Microparticles; Films; Spheres
Soft-template method	Controllable mesostructures and pore sizes; Potential for large-scale synthesis	Highly sensitive to the reaction conditions; Relatively low crystallinity	Nanofibers; Nanosheets; Microparticles; Films; Spheres; Core–shell structures
Hard-template method	Low sensitivity to the reaction conditions; Highly crystalline products	Time consuming; High cost	Nanofibers; Nanosheets; Microparticles; Films; Nanospheres; Core–shell structures; Multi-level architectures
Multiple-template method	Hierarchically and fully connected porous structures	Time consuming; High cost; Requires multiple templates	Nanofibers; Nanosheets; Microparticles; Films; Spheres; Core–shell structures; Multi-level architectures

## DIVERSE ARCHITECTURES

### Nanofibers

Nanofibers, which show many exceptional characteristics, such as large surface-to-volume ratios, quantum confinement effects, etc., have received considerable attention in recent years [17]. The large surface-to-volume ratio can significantly increase the number of surface reaction sites and modulate the catalytic activity of the surface atoms. The quantum confinement effect can change the electron and hole transport behavior [18,19]. Hierarchically mesoporous TiO_2_ nanofibers possess both merits of the porous structure and 1D morphology, which show significant potential in catalysis and energy storage.

One representative method to produce the hierarchically mesoporous TiO_2_ nanofibers is using the porous materials with 1D channels as hard templates to confine the assembly process of TiO_2_ precursors and surfactants [20]. In this case, the diameter of the 1D channel should be big enough, usually >50 nm, to accommodate the assembly process, otherwise this process may happen on the outer surface. Besides, the surface property of the 1D channel is critical because the TiO_2_ precursor  infiltrate the 1D channel driven by the capillary force.

Alternatively, by using fibrous frameworks as the shape-directing agents, hierarchically mesoporous TiO_2_ nanofibers with hollow structures can be produced [21–24]. One critical issue for this process is the surface property of the fibrous framework. For fibrous templates with abundant surface hydroxyl, the TiO_2_ precursor can uniformly form a deposit on their surface. However, for the fibrous templates with a low concentration of surface hydroxyl, the interaction between the TiO_2_ precursor and the fibrous template is weak, resulting in homogeneous nucleation of the TiO_2_ precursor, instead of heterogeneous nucleation on the surface of fibrous frameworks. Therefore, in this case, surface modification of the fibrous frameworks is critical. For example, Liu *et al.* have produced hierarchically mesoporous TiO_2_ nanofibers by using nitric acid-treated multi-wall carbon nanotubes (CNTs) as the shape-directing agent [21]. After nitric acid treatment, abundant carboxyl and hydroxyl groups are formed on the surface of CNTs, enabling the uniform deposition of the surfactant/titania-oligomer composite micelles by a layer-by-layer growth route. Another effective modification method is the introduction of a buffer layer on the surface of fibrous frameworks. For instance, after introducing a SiO_2_ buffer layer on the surface of CNTs, hierarchically mesoporous TiO_2_ nanofibers can be formed by using tetrabutyl titanate (TBOT) and cetyltrimethylammonium bromide as the precursor and pore-forming template, respectively. The SiO_2_ interlayer can not only enable the uniform deposition of titania species, but also prevent the aggregation of the TiO_2_ particles and protect the fragile fibrous structure [22].

Another efficient technique to produce nanofibers is electrostatic spinning. In this process, polymers are mixed with TiO_2_ precursors first, which can help to achieve the desired viscosity and the formation of fibers. In addition, the polymers can act as the *in situ* templates to produce porous structures [25–30]. To further synthesize TiO_2_ nanofibers with hierarchically mesoporous structures, introducing the secondary template is necessary. Wu and coworkers have developed a foaming-assisted electrospinning method for the synthesis of hierarchically mesoporous TiO_2_ nanofibers with increased specific surface areas. In this case, a foaming agent, diisopropyl azodiformate, is used as the secondary template to produce pores in the primary porous frameworks [31,32], leading to the formation of the hierarchically porous structure. However, the resultant nanofibers by this route show random distributed mesopores. On account of this, by using amphiphilic triblock copolymers [33–35] to replace the foaming agent, ordered hierarchically mesoporous TiO_2_ nanofibers can be synthesized. The advantage of the electrostatic spinning is that the obtained nanofibers possess uniform and controllable sizes. Nevertheless, the necessity of special equipment limits their wide applications.

### Nanosheets

Producing mesopores in nanosheets is an appealing endeavor in materials science, which can combine the advantages of porous structures and 2D nanostructures.

Early attempts to construct hierarchically mesoporous TiO_2_ nanosheets used flat substrates or free-standing surfaces as the structure-directing template, which is similar to the synthesis of TiO_2_ nanofibers [36–40]. Li *et al.* produced hierarchically mesoporous TiO_2_ nanosheets by using graphene oxide (GO) as the template [39]. The plentiful functional groups on the surface of GO and the slow hydrolysis and condensation rate of TBOT enable GO sheets to be conformably coated by amorphous TiO_2_ shells first. After annealing, the amorphous TiO_2_ shells would be locally crystallized; at the same time, hierarchical mesopores are generated due to the aggregation of TiO_2_ nanocrystals. This method circumvents the lattice mismatch that would inevitably arise if crystallized TiO_2_ shells with the hierarchically porous structure are directly grown on the GO sheets. To increase the regularity of the porous structure, a surfactant–template strategy was developed by using Pluronic P123 as the template [37]. The P123 template can self-assemble with the TiO_2_ precursor into the ordered mesostructure by rationally controlling the hydrolysis and condensation of titanium isopropoxide (TIPO).

**Figure 1.**
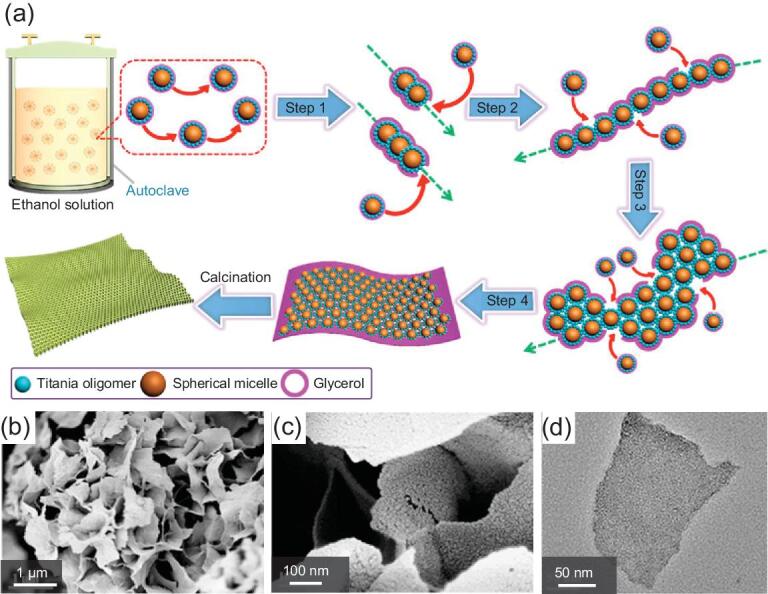
(a) Schematic illustration of the formation process for the free-standing hierarchically mesoporous TiO_2_ nanosheets via a hydrothermal-induced solvent-confined monomicelle assembly approach. The field-emission scanning electron microscopy (FESEM) (b, c) and the transmission electron microscopy (TEM) (d) images of the free-standing hierarchically mesoporous TiO_2_ nanosheets. Adapted from [41] with permission from the American Chemical Society. Copyright 2018.

Although the assembly of micelles on 2D substrates is widely used for the preparation of nanosheets, the removal of substrates can be troublesome. On account of this, Lan *et al.* have developed a solvent-confined assembly approach (Fig. 1) to synthesize free-standing TiO_2_ nanosheets with the hierarchically porous structure [41]. In this case, the stable Pluronic F127/TiO_2_ spherical monomicelles are generated first, and can be used as the subunit. The obtained monomicelles are subsequently dispersed in the mixed solvent of ethanol and glycerol, and are tightly surrounded by glycerol due to the strong hydrogen bonding. During the hydrothermal process, the assembly process of F127/TiO_2_ monomicelles occurred in a parallel direction only due to the confinement effect of glycerol networks with high viscosity. After removing the template, free-standing hierarchically mesoporous TiO_2_ nanosheets can be produced. The resultant nanosheets possess only one layer of mesopores, a high surface area of 210 m^2^ g^−1^ and a uniform thickness of 5.5 nm. Additionally, the thickness of the nanosheets can be further manipulated from 5.5 to 27.6 nm by simply tuning the precursor concentration or solvent ratio. Notably, without the confinement effect of glycerol, the monomicelles can be randomly aggregated, resulting in the formation of spherical mesostructures with the lowest surface energy.

### Microparticles

Mesoporous TiO_2_ microparticles with irregular morphologies are one of the most widely studied architectures, and can be produced by template-free, soft-, hard- and mutiple-template routes. As mentioned above, for the soft-template method, the critical issue is to rationally control the hydrolysis and condensation process of titanium precursors, thereby enabling the co-assembly with surfactant molecules. Therefore, early attempts to synthesize mesoporous microparticles were mainly carried out in acidic conditions, which could effectively mediate the hydrolysis and condensation rate of titanium precursors [42]. After that, a self-adjusted acid–base strategy was developed. In this case, titanium alkoxide and TiCl_4_ acted as a source of base and acid, respectively. The self-regulation between the acidic and basic precursors allows the control of the hydrolysis and condensation process of the titanium precursor and the cooperative assembly between the precursor and template, yielding highly ordered mesoporous TiO_2_ microparticles with tunable mesostructures [43].

However, the above-mentioned mesoporous TiO_2_ microparticles usually present amorphous or partially crystallized frameworks due to the collapse of mesostructure during template removal and framework crystallization at high temperature. The main reason for this is that commercial amphiphilic copolymers, such as the Pluronic type, are too easily decomposed, which cannot support the mesostructure at high temperature. Therefore, to obtain high-crystalline TiO_2_ microparticles, the rational design of surfactants is very important [44–46]. Wiesner and coworkers have demonstrated a cooperative assembly method that combines advantages of both soft structure-directing assemblies and hard-templating chemistries (CASH) [46]. In this method, the block copolymer (poly(isoprene-*block*-ethyleneoxide), PI-*b*-PEO) was prepared as a template. Under appropriate heating conditions, the hydrophobic PI block with *sp*^2^-hybridized carbon can convert to a sturdy amorphous carbon in the mesochannels. This *in situ-*formed carbon layer is sufficient to act as a rigid support keeping the mesostructures at temperature as high as 1000°C. The critical issue for this synthesis is that the block copolymers with a high content of *sp^2^-*hybridized carbon in the hydrophobic segments are relatively stable and could be *in situ-*converted into residual carbon at high temperature to support the mesostructure.

However, the lab-produced block copolymers show limited potential for the scalable synthesis. On account of this, in 2010, our group synthesized hierarchically mesoporous TiO_2_ microparticles with a highly crystalline framework by using sulfuric acid to carbonize the surfactants (P123) inside the mesochannels, instead of decomposition during calcination, which could support the mesostructure during the high-temperature crystallization (up to 650°C) [47]. In addition, an ethylenediamine (EN) protection route has also been demonstrated to produce thermally stable and highly crystallized hierarchically mesoporous TiO_2_ microparticles by using F127 as the template [48]. In this case, EN species with positive charges can effectively attack the surface of mesoporous TiO_2_ primary particles with negative charges, which can inhibit undesirable grain growth and phase transformation of TiO_2_ nanoparticles during the calcination process. As a result, the hierarchically mesoporous structure could be maintained even up to 700°C.

Using pre-formed and fully crystallized TiO_2_ nanocrystals to replace molecular Ti precursors as the framework building blocks is an alternative way to synthesize hierarchically mesoporous TiO_2_ microparticles with high crystallinity. The interaction between the crystallized TiO_2_ nanocrystal and surfactant is the prerequisite for this process. Therefore, the surface chemistry of the crystallized nanocrystal is of great importance, and in some cases the surface modification by removing and/or exchanging the surface ligands is necessary. Besides, the driving forces for the coassembly between nanocrystals and surfactants are mainly weak interactions, such as hydrogen bonds. Therefore, nanocrystals with a large particle size cannot work in this system [49,50]. Based on the above-mentioned principles, Milliron and coworkers have produced hierarchically mesoporous TiO_2_ microparticles by using the pre-synthesized TiO_2_ nanorods or nanospheres as the building blocks [50]. Lab-made poly (*N*,*N*-dimethylacrylamide)-*block*-polystyrene, PDMA-*b*-PS) was selected as the structure-directing agent because the PDMA can mimic the dynamic adsorption interaction of dimethylformamide at the surface of TiO_2_ nanocrystals. Hence, the hierarchically mesoporous TiO_2_ with a high surface area and fully crystalline framework can be produced.

**Figure 2.**
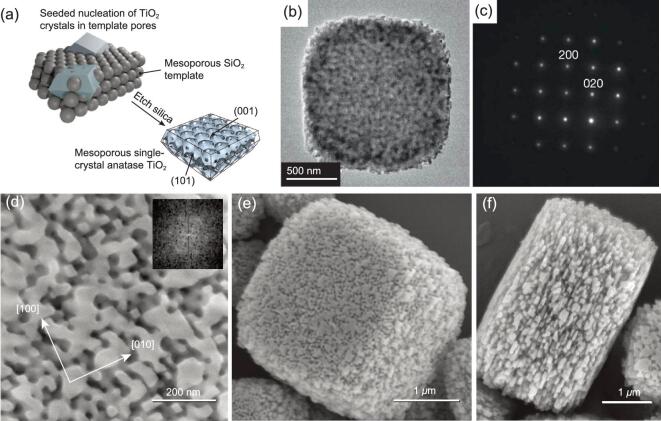
(a) Schematic illustration of the synthesis of a hierarchically mesoporous TiO_2_ single crystal via a seeded nucleation and growth method by using monodispersed SiO_2_ spheres as the hard template. The TEM (b), electron diffraction Laue pattern (c), and scanning electron microscopy (SEM) (d–f) images of the hierarchically mesoporous TiO_2_ single crystals. Adapted from [51] with permission from Nature Publishing Group. Copyright 2013.

Alternatively, the hard-template route is a facile and general way to synthesize hierarchically mesoporous TiO_2_ microparticles crystalline frameworks [51–53]. For example, Crossland *et al.* have reported a general seeded nucleation and growth method to synthesize the mesoporous anatase single-crystal with a high surface area (70 m^2^ g^−1^) by using monodisperse silica spheres as the template (Fig. 2) [51]. The results suggest that ‘seeding’ the template with microscopic nucleation sites is vital for the confining growth of single crystal TiO_2_, which directly overwhelms the homogeneous nucleation.

**Figure 3.**
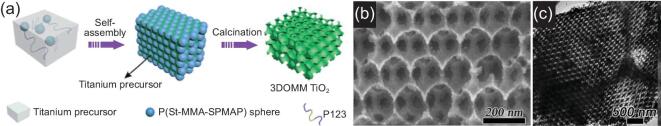
(a) Schematic illustration of the synthesis of ordered hierarchically mesoporous TiO_2_ microparticles by using P(St-MMA-SPMAP) spheres and P123 as hard and soft templates, respectively. SEM (b) and TEM (c) images of hierarchically mesoporous TiO_2_ microparticles. Adapted from [61] with permission from the Royal Society of Chemistry. Copyright 2014.

However, in the above cases, the secondary pores in the hierarchically mesoporous frameworks are generated by the accumulation of nanoparticles, which show uncontrollable sizes and distribution. To further increase the size and regularity of the secondary pores, the multiple-template route, i.e. a combination of soft- and hard-templating, was developed [54–56]. For example, hierarchically mesoporous TiO_2_ materials have been produced by removal of SiO_2_ in the mesoporous crystalline TiO_2_–SiO_2_ nanocomposites. In this case, highly ordered 2D hexagonal mesoporous TiO_2_–SiO_2_ nanocomposites with variable Ti/Si ratios were firstly obtained by co-assembly of TIPO, tetraethyl orthosilicate (TEOS) and Pluronic P123. Utilizing sodium hydroxide (NaOH) as an etchant to remove SiO_2_ can produce secondary pores. The final products, possessing hierarchical mesopores, are highly connected by uniform intrawall mesopores while retaining mesostructural integrity and regularity. Besides, the size of the secondary pore can be fine-tuned from 0.9 to 4.8 nm by changing the crystallization temperature and Ti/Si ratio [57,58]. To better control the hierarchically porous structures, colloidal crystals constructed by SiO_2_ or polymer nanospheres were introduced as the hard templates [59,60]. For example, Su and coworkers have synthesized ordered hierarchically mesoporous TiO_2_ microparticles by using P(St-MMA-SPMAP) spheres and P123 as the hard and soft templates, respectively (Fig. 3) [61]. By this route, the pore size, pore structure and wall thickness can be well controlled. Close-packing of the P(St-MMA-SPMAP) spheres with uniform size is crucial for the formation of ordered hierarchically porous structures.

### Films

Mesoporous TiO_2_ films are very useful for the fabrication of devices such as dye-sensitized solar cells, electrochromic devices, antifogging, antibacterial, self-cleaning coatings and many others. The most widely used method to synthesize mesoporous TiO_2_ films is the evaporation-induced self-assembly (EISA) approach. To date, various kinds of mesoporous TiO_2_ films have been fabricated by employing different ionic and non-ionic surfactants, like commercially available non-ionic triblock copolymers of the Pluronic family with the general formula EO*_x_*PO*_y_*EO*_x_* [62–64]. However, those resultant mesoporous TiO_2_ films exhibit poor thermal stability, and mesostructures would collapse at high temperature. Moreover, the sizes of the mesopores are usually less than 10 nm. To circumvent the above problems, a series of lab-made copolymers [65–67], which contain a high content of *sp^2^-*hybridized carbon, were synthesized to produce highly crystalline mesoporous TiO_2_ films with tunable mesostructures and pore sizes. For instance, Feng *et al*. have reported a ligand-assisted EISA method to produce mesoporous TiO_2_ films on various substrates via using PEO-*b*-PS as the template [67]. Acetylacetone was utilized as the ligand to reduce the hydrolysis and condensation rate of the titanium precursor, enabling the cooperative assembly with the surfactant. Besides, the PS segment containing *sp^2^-*hybridized carbon can generate carbon residues as a protection layer to support the TiO_2_ framework during the pyrolysis, thereby sustaining the mesostructure. The obtained TiO_2_ films possess a monoclinic mesostructure distorted from a (110)-oriented primitive cubic structure, and the thickness can be well controlled from 150 nm to several micrometers by changing the parameters of dip-coating.

The orientation of mesopores is a crucial parameter of mesoporous films. In most reported cases, the alignment of mesopores is parallel to the substrate surface due to the principle of the lowest energy. Actually, mesopores with vertical alignment are highly desired because such structures can improve the transportation of electrons, ions and fluids in the films, which is especially important for solar cells, fuel cells and separation technologies [68]. To this end, Wu *et al.* have reported a unique structural transformation process to prepare mesoporous TiO_2_ films with perpendicular channels. In this case, mesoporous TiO_2_ films (space group: 3D hexagonal) with the *c*-axis perpendicular to the substrate are firstly synthesized [69]. Subsequently, the hexagonal structure is transformed into arrays of TiO_2_ pillars with open-spaced, perpendicular and continuous porosity after calcination at 400°C. The structural transformation is mainly caused by the large contraction (40%) of the mesostructure along the direction perpendicular to substrates during the thermal process. To further improve the permeability and control the orientation of mesoporous TiO_2_ films, Shan *et al*. have developed a facile hot air flow-assisted route to synthesize the mesoporous TiO_2_ films with vertical orientation [70]. The hot gas results in the quick evaporation of solvents on the solution surface, leading to a concentration gradient with the highest surfactant concentration at the solution surface. At the same time, cylindrical micelles are formed at the liquid–vapor interface. In this process, the shear force generated by the air flow and the upward growth induced by solvent evaporation make a joint effect on the orientation of mesochannels. As a result, the orientation of the mesopores can be precisely controlled by tuning the incident angle of the air jet to the surface plane and the rate of solvent evaporation.

Apart from controlling the orientation of mesopores, construction of hierarchical mesopores with large pore sizes in films is another way to improve the transportation of guest molecules. The multiple-template route is an effective way to produce the hierarchical mesopores in the films, too [71]. Besides, by inducing the phase separation of the precursors and surfactants during the assembly process, the hierarchically mesoporous TiO_2_ films can also be fabricated. Polyethylene glycol (PEG) is one of the most used reagents to induce the phase separation because titania oligomers can be adsorbed on PEG by hydrogen bonds [72], resulting in the formation of secondary pores in the hierarchically mesoporous TiO_2_ frameworks.

### Spheres

With the highest symmetry, spherical mesoporous TiO_2_ materials have stimulated intensive research for applications in energy storage, photocatalysis and environment recombination. In order to achieve spherical morphology, various hard templates, such as mesoporous nanospheres and reverse opals, have been initially adopted to confine the sol–gel self-assembly process [73,74].

Soft-template methods for the synthesis of mesoporous TiO_2_ nanospheres have been successively proposed to simplify the hard-template route. It is vital to develop a facile and reproducible sol–gel method to prepare monodisperse and uniform mesoporous TiO_2_ nanospheres. Chen *et al*. have creatively combined the sol–gel process with a solvothermal treatment in the presence of hexadecylamine (HDA) as the structure-directing agent [75]. The lipophilic interactions between the long-chain alkyl groups in alkylamine can drive the self-assembly process of the hydrolyzed Ti species/oligomers to produce monodisperse nanospheres. This strategy is facile to fabricate hierarchically mesoporous TiO_2_ nanospheres [76]. To improve the universality and repeatability of this route, Zhu *et al*. have reported a double surfactant-directed assembly method to prepare monodisperse hierarchically mesoporous TiO_2_ nanospheres by using *n*-dodecylamine (DDA) and Pluronic F127 as templates [77,78]. In this process, F127 and DDA can assemble into cooperative spherical micelles in the alcohol/water solution. After interacting with the hydrolyzed titanium species, a stronger lipophilic interaction can be formed between the resultant spherical micelles/oligomer composites, which could promote the self-assembly of those spherical micelles/oligomer composites, leading to a fast phase separation process to form small-sized TiO_2_ nanospheres. Besides, by varying the concentration of surfactants, the diameter sizes of hierarchically mesoporous TiO_2_ nanospheres can be simply changed from 50 to 250 nm.

The aerosol-assisted self-assembly (spray drying) process is another very promising strategy aimed at the low-cost, and scalable synthesis of mesoporous spheres [79–81]. Pal *et al.* have introduced a relatively low drying temperature (170°C) and an additional ultrasonication route to synthesize hierarchically mesoporous TiO_2_ spheres by using Pluronic P123 as the soft template [80]. During post-ultrasonication treatment, the low-polymerized TiO_2_ frameworks undergo further hydrolysis and condensation, thereby transforming into an aggregate of small TiO_2_ grains and resulting in the formation of the hierarchically porous structure [82]. The spray-drying technique is a feasible strategy for controlling the diverse surface morphology of hierarchically mesoporous TiO_2_ spheres as well. For example, by controlling drying temperature, the droplet jet dispersed in the drying chamber can rapidly dry, resulting in the formation of hierarchically mesoporous TiO_2_ spheres with a wrinkled surface [81].

**Figure 4.**
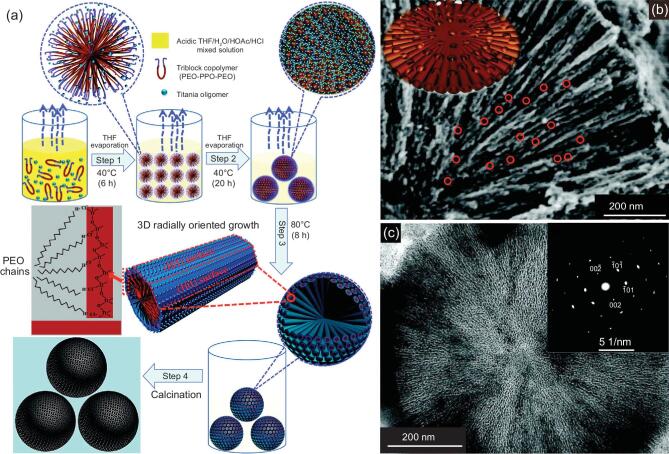
(a) Schematic representation of the formation process of hierarchically mesoporous TiO_2_ microspheres with a single-crystal-like pore wall through EDOA approach. (b) The SEM image of a single ultramicrotomed, radially oriented hierarchically mesoporous TiO_2_ microspheres. Inset: structure models for the radially oriented channels with interchannel pores. (c) The TEM image of a single ultramicrotomed, hierarchically mesoporous TiO_2_ microsphere. Inset: the selected-area electron diffraction (SAED) pattern taken from the cylindrical pore bundles region with [010] incidence. Adapted from [83] with permission from the American Association for the Advancement of Science. Copyright 2015.

The above-mentioned strategies suffer from a common problem that because of the spontaneously random assembly of micelles, these materials have polycrystalline pore walls that are generally irregularly oriented in space, which adversely affects the transport of electrons and ions. To solve this problem, Liu *et al*. have demonstrated an evaporation-driven oriented assembly (EDOA) approach to synthesize hierarchically mesoporous TiO_2_ spheres with single-crystal-like anatase walls and radially oriented mesopores  (Fig. 4a) [83]. At the first step, evaporation of tetrahydrofuran (THF) at 40°C caused the formation of Pluronic F127–TiO_2_ oligomer spherical composite micelles. The evaporation of residual THF and the hydrolysis solvent, such as *n*-butyl alcohol, proceeded more vigorously at 80°C, which drove the initially formed spherical micelles to grow along the free radial and restricted tangential direction within the microspheres. In this process, the crystallographic orientation of the nanocrystal building blocks can be controlled by the slow evaporation and confinement of the triblock copolymer hydrophilic boundary (Fig. 4b and c). Later, by adjusting the evaporation temperature, ellipsoid hierarchically mesoporous TiO_2_ mesocrystals could be obtained

[84]. More recently, we have developed a coordination-mediated self-assembly method to precisely control the phase composition of the resultant hierarchically mesoporous TiO_2_ spheres [85]. A low concentration of HCl favors the nucleation and growth of the anatase phase with more edge-shared [TiO_6_] octahedra, while the corner-shared process with one dehydration reaction between [TiO_6_] octahedr occurs more easily under the condition with high-concentration HCl, which is beneficial for the nucleation and growth of rutile [86]. More interestingly, after being combined with the hydrothermal treatment, an unprecedented type of dehiscent hierarchically mesoporous TiO_2_ rutile spheres with the radial mesopore channels can be obtained. The main reason for the formation of the dehiscent architecture is that the solvents, including *n*-butanol from TBOT hydrolysis as well as a spot of residual THF and water, in the pre-calcinated spheres can generate high vapor pressure during the hydrothermal process, resulting in the splits on the surface of spheres [87].

**Figure 5.**
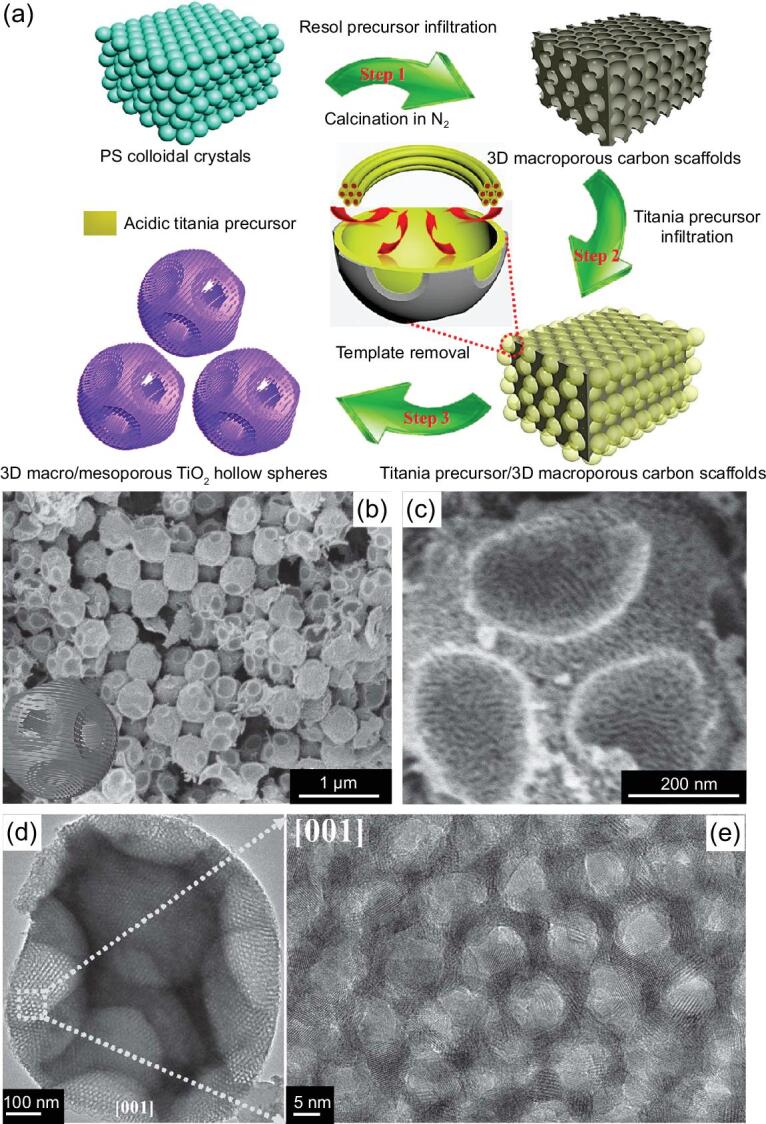
(a) Schematic representation of the formation process for hierarchically macro/mesoporous TiO_2_ hollow microspheres by using 3DOM and Pluronic F127 as the hard and soft template, respectively. The SEM (b, c), TEM (d) and high resolution transmission electron microscopy (HRTEM) (e) images of the hierarchically macro/mesoporous TiO_2_ hollow microspheres. Adapted from [88] with permission from Wiley-VCH. Copyright 2016.

To produce hierarchically mesoporous TiO_2_ spheres with ordered porosity, Liu *et al*. have reported the synthesis of highly crystalline hierarchically ordered macro/mesoporous TiO_2_ hollow microspheres through using the multiple-template route (Fig. 5) [88]. In this case, 3D-ordered macroporous carbon scaffolds (3DOMC, inverse opal structures) with uniform cavities and Pluronic F127 were used as hard and soft templates, respectively, to produce ordered mesopores and hollow structures. The resultant hierarchically macro/mesoporous hollow microspheres possess controllable ordered mesostructure symmetry (hexagonal *p6mm* or cubic *Im3m*) and highly crystalline anatase frameworks. Later, similar hierarchically ordered macro/mesoporous TiO_2_ spheres were synthesized by using 3DOMC and Pluronic P123 as templates by Luo and coworkers [89]. Interestingly, in Luo's work, through tuning the relative amount of the precursor solution in the 3DOMC (partially or completely filling), hierarchically mesoporous TiO_2_ particles with unique morphologies, such as hemispheres, can be produced.

### Core–shell structures

Core–shell structured nanomaterials provide a platform for integrating multiple building blocks into a single functional system, exhibiting enhanced or new physical and chemical properties that are not available in the isolated components [90,91].

In 2012, Li *et al.* for the first time, reported a versatile kinetics-controlled coating method for the preparation of core–hierarchically mesoporous TiO_2_ shell nanomaterials in a pure ethanol system with ammonia as the catalyst [92]. In this system, the concentration of ammonia that can mediate the reaction kinetics of TBOT is critical. Under the condition of a low concentration of ammonia, the reaction rate is slow, resulting in the heterogeneous nucleation on the core surface and then the formation of uniform shells. However, a high concentration of ammonia leads to a high reaction rate, leading to the simultaneous heterogeneous and homogeneous nucleation. As a result, large and aggregated nanoparticles are formed. Notably, this method is very straightforward, versatile and can be widely used for the synthesis of various core– mesoporous TiO_2_ shell nanomaterials with tunable shell thickness [93].

Later, Lou and coworkers developed a universal cooperative assembly method for coating TiO_2_ shells with the hierarchically porous structure on various cores with different compositions and morphologies [94]. In this work, HDA was used as the template. The amino groups of HDA molecules can participate in hydrogen-bonding interactions with TIPO hydrolysis products to form inorganic–organic composites, which can be coated on the nanoparticles. Besides, the long hydrophobic carbon chains of HDA self-organize into rod-like micelles that can produce pores in TiO_2_ domains. This method is simple and universal, and can rapidly produce hierarchically mesoporous TiO_2_ shells on various inorganic, organic and inorganic–organic composite materials, including silica, metals, metal oxides, organic polymers, carbon-based and metal–organic framework (MOF) nanomaterials. Moreover, this strategy also provides a versatile platform toward the fabrication of various TiO_2_-based novel and hierarchical nanostructures, including hollow and yolk–shell structures with tailored cavity sizes, shell thicknesses tailored cavity sizes and shell thicknesses.

**Figure 6.**
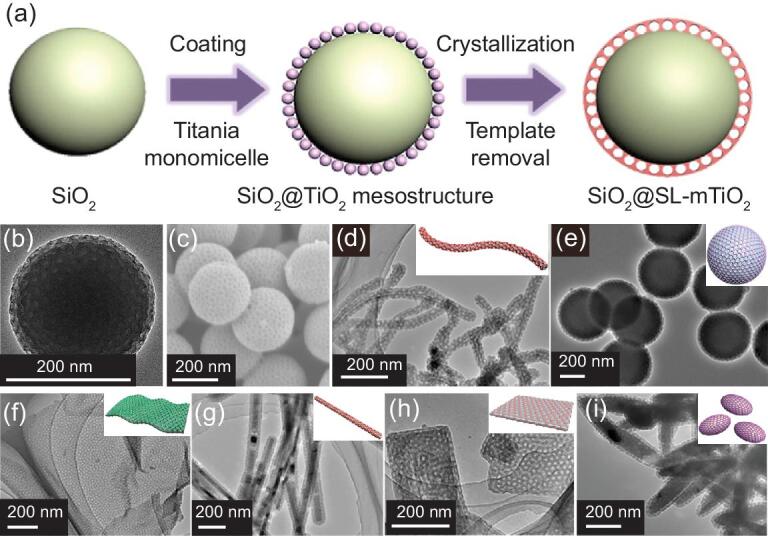
(a) Schematic illustration of the preparation of single-layer (SL) hierarchically TiO_2_ mesopore-coated core–shell structures. TEM (b) and FESEM (c) images of the SiO_2_@SL-mTiO_2_ core–shell nanostructures. TEM images of different nanomaterials from 1D to 3D with coated single-layered hierarchically TiO_2_ mesopores: (d) carbon nanotubes, (e) carbon nanospheres, (f) graphene oxides, (g) CdS nanowires, (h) ZnS nanosheets, (i) α-Fe_2_O_3_ ellipsoids. The insets in (d–i) are the corresponding structural models. Adapted from [95] with permission from Elsevier. Copyright 2019.

To better control the sizes of mesopores, our group has demonstrated a general confined interfacial monomicelle assembly approach for coating mesoporous TiO_2_ shells (Fig. 6) [95]. In this process, the F127/TiO_2_ composite monomicelles are obtained first, which can act as the subunit. After redispersal into the solution containing ethanol and glycerol, the monomicelles tend to collide and attach on the surface of functional cores by a side by side packing manner due to the confinement effect of glycerol and the shear force generated by stirring, resulting in the formation of an ordered structure. After removing the template by calcination, mesoporous shells can be produced. This assembly process shows precise controllability and great versatility, endowing the coated TiO_2_ layers with highly tunable thickness, mesopore sizes, and switchable coated surfaces. Furthermore, the accurate controllability of such a confined assembly process enables the formation of TiO_2_ shells from mono- to multilayers (up to five layers) of mesopores, and the mesopore size can be manipulated from 4.7 to 18.4 nm by tuning the amount of swelling agent. This method is highly reproducible and reliable and provides a deep insight into rational design and precise synthesis.

After removing the interior core in the core-shell nanomaterials mentioned above, hollow hierarchically mesoporous structures can be produced [96–99]. However, in some cases, the pristine morphologies cannot be maintained because of the structural collapse during the removal of interior cores. To circumvent this problem, Yin and coworkers have developed a silica-protected calcination process, which relies on an additional silica coating to limit the structural rearrangement of TiO_2_ [100], but the resultant material shows poor crystallinity. To better control the nanoscale crystallinity in the hierarchically mesoporous TiO_2_ shells, the same group has developed a novel partial etching and re-calcination process. The partial etching step produces a small gap between SiO_2_ and TiO_2_ layers, which allows space for the further growth of TiO_2_ into large crystal grains. The re-calcination process leads to highly crystallized TiO_2_, which maintains the hierarchically porous structure due to the protection of the partially etched outer silica layer [101].

Multi-shelled mesoporous architectures manifest remarkable superiority toward some specific applications [102,103]. Ren *et al.* have developed a sequential templating approach to synthesize multishelled mesoporous hollow TiO_2_ nanospheres with hierarchical pores by using carbonaceous nanospheres and TiCl_4_ as sacrificial templates and precursors, respectively [104]. The adsorption of Ti species in carbonaceous nanospheres is a prerequisite for the successful synthesis. The concentration of precursor plays an essential role in this route: too low or too high results in the formation of electronegative anions [Ti_n_O_4n_]^4n−^ or [Ti(OH)_n_Cl_6−n_]^2−^, respectively, which are the repulsion with negatively charged carbonaceous nanosphere templates. Moreover, by changing the calcination parameters, the shell numbers, shell thicknesses and the spacing between shells of the multi-shelled structure can be well controlled. After that, by regulation of the reaction conditions (precursors, post-hydrothermal treatment, calcination, etc.), a series of multi-shelled hierarchically mesoporous TiO_2_-based heterostructures such as SrTiO_3_–TiO_2_ [105], anatase–TiO_2_(B) [106] and TiO_2-x_–TiO_2_ [107], were synthesized by the same group.

### Multi-level architectures

Bio-templating is an efficient and universal approach to synthesize hierarchically mesoporous materials with mutli-level architectures. The main advantage of this method is that a variety of natural materials with low-cost and environment-friendly properties can act as templates. Generally, the synthetic process for the bio-template method involves: (i) after absorbing the titanium precursors by the cell walls of bio-templates through capillary adsorption, an amorphous TiO_2_ replicated from bio-templates can be formed, and (ii) the natural templates are removed by calcination; at the same time, hierarchically mesoporous TiO_2_ materials with mutli-level architectures with similar characteristics to the pristine natural templates can be obtained.

Butterfly wings, which display diverse colors and patterns due to their periodic surface scales, are representative bio-templates and can be used for the synthesis of hierarchically porous materials with unique properties [108,109]. By using butterfly wings of different breeds, a variety of hierarchically mesoporous TiO_2_ materials with quasi-honeycomb-like shallow concavities and cross-ribbing structure can be produced [110]. Apart from butterfly wings, recently, a series of bio-templates such as bacteria [111], cellulose [112] and yeast [113] have been used for the synthesis of hierarchically mesoporous TiO_2_ materials with multi-level architectures. However, it is difficult to precisely control the resultant structures at nanoscale due to the non-uniformity of bio-templates.

**Figure 7.**
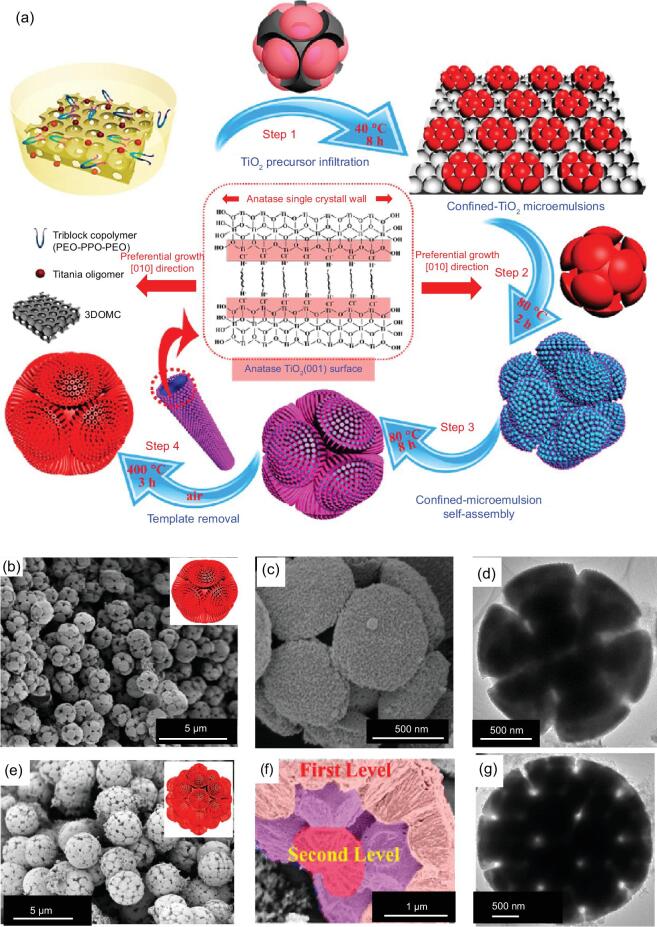
(a) Schematic representation of the formation process of hierarchically mesoporous TiO_2_ bouquet-posy-like superstructures through confined-microemulsion self-assembly process. FESEM (b, c) and TEM (d) images of the Level-1 hierarchically mesoporous TiO_2_ superstructure. FESEM (e, f) and TEM (g) images of the Level-2 hierarchically mesoporous TiO_2_ superstructure. Adapted from [114] with permission from the American Chemical Society. Copyright 2016.

To address this issue, Liu *et al.* have reported a confined microemulsion self-assembly approach to synthesize an unprecedented type of 3D highly ordered hierarchically mesoporous TiO_2_ with bouquet-posy-like architectures (Fig. 7) [114]. 3DOMC was used as the template, in which F127/ TiO_2_ oligomer micelles separate into uniform microemulsion droplets due to the surface tension. The Level-1 hierarchically mesoporous superstructures, which consist of one spherical core and 12 symmetric satellite hemispheres, resulted from an interface tension-induced shrinkage procedure. The THF evaporation can increase the interface tension force, which tends to pull the formed microemulsion droplets to complete filling of the center macropores of 3DOMCs, leading to the formation of a spherical core at the center of the microemulsion droplets. At the same time, the volume of the microemulsion droplets in macropores is dramatically decreased with the continuous evaporation, and the shrinkage of microemulsion droplets occurs, making one spherical core coupled with 12 satellite hemispheres via 12 connected macropore windows of the 3DOMC scaffolds. Notably, by increasing the size or content of impregnated TiO_2_ precursor emulsion droplets, a more complex and asymmetric superstructure with 13 spherical cores and up to 44 symmetric satellite hemispheres can also be well manipulated.

## APPLICATIONS

Owing to its unique physical, chemical and optical properties, hierarchically mesoporous TiO_2_ materials have been widely utilized in various applications concerning the major three challenging themes in the 21st century, including energy, environment and health. In this section, we highlight the applications of hierarchically mesoporous TiO_2_ materials in the energy and environment, including photocatalytic degradation pollutants, photocatalytic fuel production, photoelectrochemical cells, catalyst support, lithium-ion batteries and sodium-ion batteries.

### Photocatalytic degradation of pollutants

Hierarchically mesoporous TiO_2_ materials are of great interest in the area of photocatalytic degradation of pollutants due to their high surfaces and large pore volumes. High surface areas can provide a large number of active sites for adsorption and reaction of reactants. Besides, large pore volumes can facilitate the transport of chemicals in the bulk of TiO_2_ materials, enabling the accessibility of active sites. When being used as photocatalysts for removal of acetaminophen in water, the decomposition rate of the mesoporous TiO_2_ microspheres is significantly faster than that of nonporous commercial P25 due to their high surface areas [115]. Moreover, the photocatalytic performance can be further improved by the fabrication of hierarchically porous structures [74]. However, the activities of TiO_2_ materials are greatly limited by the wide band gap and low quantum efficiency.

Heteroatoms doping is an effective modified way to introduce additional extrinsic electronic levels in the energy band gap, thereby promoting light absorption. Recently, a series of heteroatoms dopants, including metal and non-metal atoms, have been reported to enhance the performances of TiO_2_ materials [116,117]. For example, compared with the undoped form, iodine-doped hierarchically mesoporous TiO_2_ can remove toxic organic pollutants more effectively  [117]. Co-doping with two heteroatoms can further improve the activity of hierarchically mesoporous TiO_2_. The C,N-co-doped hierarchically mesoporous TiO_2_ material shows a better visible-light photocatalytic activity on the degradation of ibuprofen than single heteroatom doped and undoped forms [118]. However, for doped TiO_2_, the lattice defects resulted from the dopants can unavoidably introduce new charge carrier trapping and recombination centers, which have negative effects on the photocatalytic performance.

**Figure 8.**
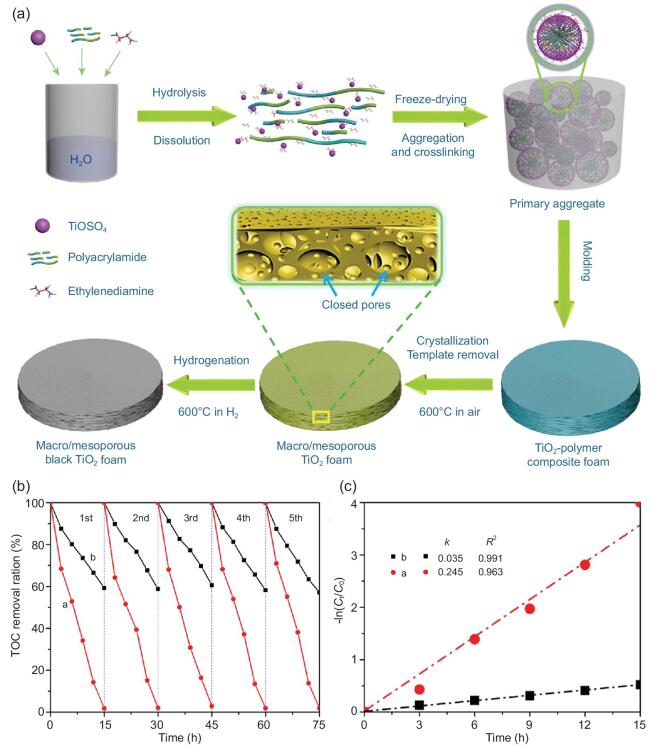
(a) Schematic illustration of the formation process of self-floating hydrogenated hierarchically macro-mesoporous TiO_2_ foams. The recycle (b) of hydrogenated hierarchically macro-mesoporous TiO_2_ foams (red line) and hydrogenated P25 (black line) for the TOC removal of hexadecane under AM 1.5 without stirring and the corresponding variation (c) of −ln(*C*_t_/*C*_o_) vs. AM 1.5 irradiation (*C*_t_ and *C*_o_ are the corresponding degradative concentration and initial concentration of hexadecane, respectively). Adapted from [120] with permission from Elsevier. Copyright 2017.

Hydrogenated TiO_2_ materials, without the introduction of unwanted carrier recombination centers from dopants, were firstly reported by Mao and coworkers in 2011 [119] to decrease the band gap of TiO_2_. Zhou *et al*. have reported the band gaps of hydrogenated hierarchically macro/mesoporous TiO_2_ materials is significantly smaller than that of the pristine one [120]. Moreover, the resultant materials show excellent solar-driven photocatalytic activity and long-term stability for complete mineralization of floating insoluble hexadecane. In addition, the photocatalytic reaction apparent rate constant *k* is 7 times higher than that of commercial P25 under AM 1.5 irradiation (Fig. 8). Later, by using urea as a nitrogen resource, the same group further synthesized hydrogenated hierarchically mesoporous TiO_2_ doped by N, which can degrade 96% methyl orange under visible light in 180 min [121].

Construction of hierarchically mesoporous TiO_2_ nanocomposites, such as metals–TiO_2_ [122,123], metal sulfides–TiO_2_ [124] and carbon–TiO_2_ [125] is also a promising way of promoting the activities. The intimate contact of TiO_2_ and other materials leads to the formation of heterojunctions, which remarkably favors the separation of photogenerated electrons and holes. Jiang and coworkers have fabricated the hierarchically mesoporous TiO_2_/graphene composites for degrading methyl blue [125]. The apparent rate constant for the hierarchically mesoporous TiO_2_ films with graphene can up to be 0.071 min^−1^, almost 1.6 times that for hierarchically mesoporous TiO_2_ films without graphene.

### Photocatalytic fuel production

Developing renewable energy epitomizes one of the major scientific challenges for the 21st century. One of the cleanest approaches is photocatalytic fuel production (H_2_ generation, CO_2_ reduction, etc.). Recently, various advanced TiO_2_ nanostructures have been developed to achieve this goal. Here we summarize some key progress regarding hierarchically mesoporous TiO_2_ materials for photocatalytic fuel production.

#### Photocatalytic H_2_ generation

Photocatalytic water splitting has been proven to be an ideal, sustainable, eco-friendly and inexhaustible approach to producing H_2_ without environmental pollution. Porous TiO_2_ materials are some of the most investigated photocatalysts for H_2_ generation due to their low cost, abundance and environmental benignity. Lasa and coworkers have demonstrated that hierarchically mesoporous TiO_2_ shows excellent photocatalytic performance for H_2_ production [126]. The quantum yield is estimated to be 22.6%, which is significantly higher than that of nonporous commercial P25. However, as mentioned above, for real applications, the performance of hierarchically mesoporous TiO_2_ is greatly limited by its wide band gap and low efficiency of charge separation.

Surface hydrogeneration of TiO_2_ is an effective way because hydrogeneration can lead to the formation of defects, which can increase the solar-light adsorption and improve the charge separation and transportation [53]. Zhou *et al.* for the first time, have prepared hydrogenated mesoporous TiO_2_ materials for catalytic H_2_ generation [127]. The photoresponse of the resultant hydrogenated TiO_2_ can extend from UV light to visible and infrared light regions. As a result, the obtained materials show excellent photocatalytic hydrogen generation performance with a rate of 136.2 μmol h^−1^ by using Pt and methanol as the co-catalyst and sacrifice reagent, respectively, which is almost twice as high as that of the pristine one (76.6 μmol h^−1^). By filtering out incident light with wavelengths shorter than ∼400 nm, the resultant materials still exhibit a good photocatalytic activity, while the pristine one shows no visible-light activity.

**Figure 9.**
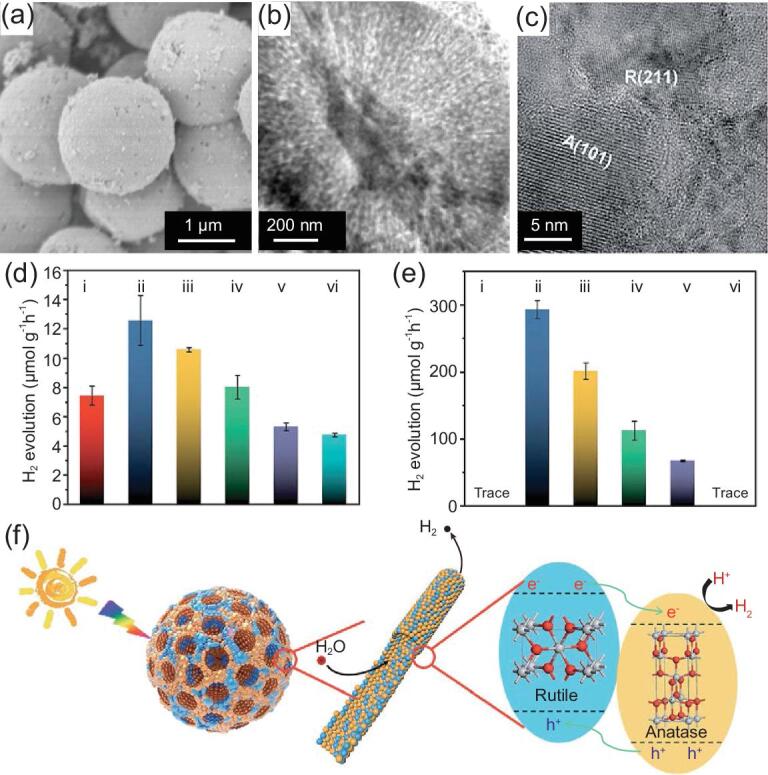
FESEM (a), TEM (b) and HRTEM (c) images of hierarchically mesoporous TiO_2_ microspheres with anatase/rutile phase junctions. H_2_ evolution rates under (d) AM 1.5 G and (e) visible-light (λ > 400 nm) of the (i) hierarchically mesoporous anatase, (ii) hierarchically TiO_2_ (anatase:rutile = 77:23), (iii) hierarchically mesoporous TiO_2_ (anatase:rutile = 60:40), (iv) hierarchically mesoporous TiO_2_ (anatase:rutile = 40:60), (v) hierarchically mesoporous rutile, and (vi) commercial P25 nanoparticles. (f) Schematic diagram illustrating the process of H_2_ evolution across the hierarchically mesoporous TiO_2_ microspheres with phase junctions. Adapted from [85] with permission from the Royal Society of Chemistry. Copyright 2019.

In addition, construction of TiO_2_ phase junctions can promote the separation of charges and holes significantly, thereby improving performances of TiO_2_ materials. For example, hierarchically mesoporous microspheres with an anatase/rutile ratio of 77:23 have shown excellent photocatalytic performance with an H_2_ generation rate up to 12.6 μmol h^−1^ g^−1^—significantly higher than that of hierarchically mesoporous single-phase microspheres and commercial P25 [85]. Notably, the hierarchically mesoporous microspheres possess excellent visible-light activity, too. The H_2_ generation rate can be up to 293 μmol g^−1^ h^−1^ after cutting off the UV light shorter than 400 nm (Fig. 9). After introducing the defects with controllable distribution to the hierarchically mesoporous TiO_2_ microspheres with anatase-rutile phase junctions, the H_2_ generation rate can be further improved, reaching 21.3 μmol h^−1^ g^−1^ and 852 μmol g^−1^ h^−1^ under AM 1.5 G and visible light, respectively [128]. Single-crystals also present great potential for photocatalytic H_2_ generation. Hierarchically mesoporous rutile TiO_2_ microspheres with the single-crystal-like wall and dehiscent architecture can produce hydrogen gas steadily at ∼12.2 μmol h^−1^ g^−1^, almost three times higher than that of commercial P25 [87].

#### Photocatalytic CO_2_ reduction

The photocatalytic CO_2_ reduction into hydrocarbon fuels is a promising approach for the direct conversion of CO_2_ to value-added chemicals (CO, methane, methanol, etc.) by sunlight. This process is much more complicated than water splitting for the following reasons: (i) reduction of CO_2_ requires a higher energy input for breaking the O=C=O double bond; (ii) the side reaction happens simultaneously during CO_2_ reduction, resulting in the low selectivity for target products [129]. It has been reported that hierarchically mesoporous TiO_2_(B) shows excellent performance in catalyzing CO_2_ to methanol and methane [130]. However, when using TiO_2_ materials as CO_2_ reduction photocatalysts, some issues, like wide band gap, low quantum efficiency and weak interaction between the TiO_2_ surface and CO_2_, should be addressed.

One common and effective way is doping. Ye and coworkers have prepared cobalt (Co)-doped hierarchically mesoporous TiO_2_ for catalytic CO_2_ reduction [131]. The introduction of Co ions can change the location of the conduction band and valence band of TiO_2_, leading to visible-light absorption. Thus, such designed material exhibits a high activity for the reduction of CO_2_. By varying the molar ratio of Co/Ti, the optimal generation rate of CH_4_ can be increased to 0.258 μmol g^−1^ h^−1^.

**Figure 10.**
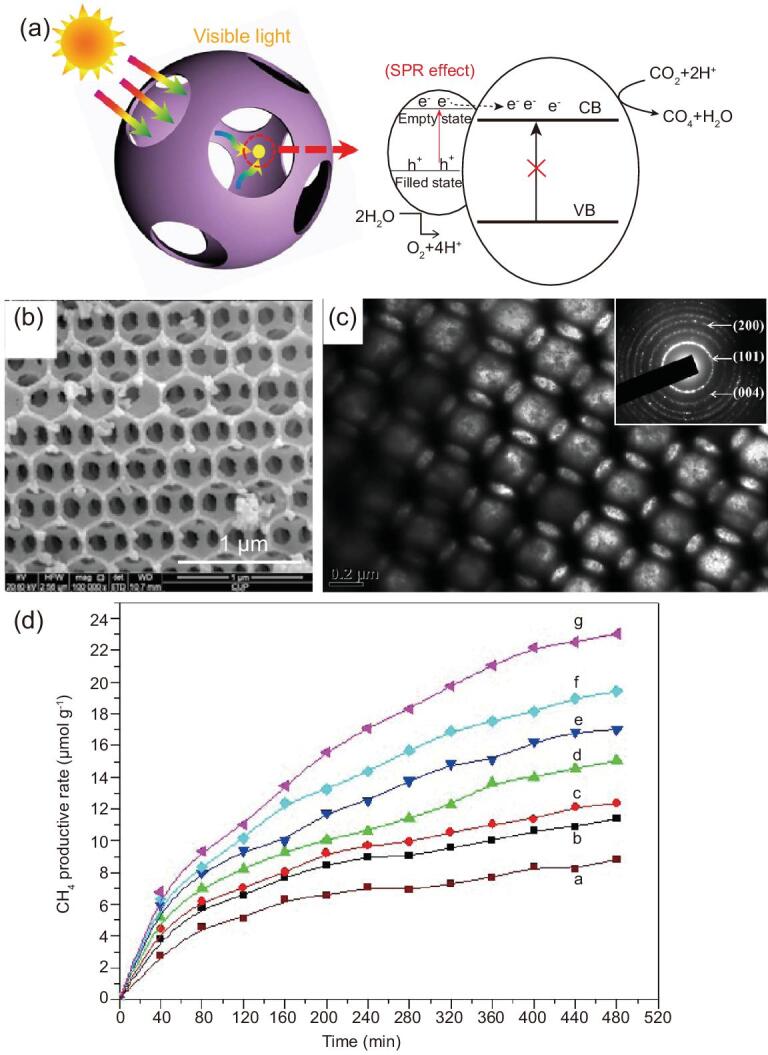
(a) The mechanism for photocatalytic reduction of CO_2_ with H_2_O to methane over hierarchically porous Au/TiO_2_ catalysts. SEM (b) and TEM (c) images of hierarchically porous TiO_2_. (d) CH_4_ production amounts over P25, hierarchically mesoporous TiO_2_ and Au/TiO_2_ catalysts: a, P25; b, TiO_2_; c, TiO_2_ with 0.5 wt% Au; d, TiO_2_ with 1 wt% Au; e, TiO_2_ with 2 wt% Au; f, TiO_2_ with 4 wt% Au; g, TiO_2_ with 8 wt% Au. Adapted from [136] with permission from Elsevier. Copyright 2015.

Additionally, fabrication of heterostructures is optional. Cu_2_O-modified hierarchically mesoporous TiO_2_ hollow spheres have been demonstrated to catalyze the conversion of CO_2_ to CH_4_ under visible-light [132]. Cu_2_O is a typical p-type semiconductor with a narrow optical band gap of ∼2.1 eV. When combined with TiO_2_, the high separation efficiency of photo-induced charges and holes can be achieved due to the formation of *p–n* junctions between Cu_2_O and TiO_2_. Thus, the overall CH_4_ generation rate can be up to 0.16 μmol g^−1^ h^−1^. In addition to metal oxides–TiO_2_ heterostructures, construction of noble metals–TiO_2_ heterostructures can improve the photocatalytic performance of CO_2_ reduction, too [133,134]. In this system, the formation of Schottky junctions between noble metals and TiO_2_ can improve the separation and transportation of charge carriers, and the local surface plasmon resonance (LSPR) effect of noble metals can extend the photoresponse of TiO_2_ from the UV to the visible-light region. For example, Tu *et al.* have prepared the Au@mesoporous TiO_2_ yolk–shell spheres for plasmon-induced photocatalytic reduction of CO_2_ [135]. The resultant materials show high generation rates of CH_4_ and C_2_H_6_ of 2.52 and 1.67 μmol g^−1^ h^−1^, respectively, indicating that the introduction of Au can accelerate multiple electron/hole reactions, and thus generate more valuable high-grade carbon species via an enhanced C–C coupling reaction. Jiao *et al.* have designed 3D hierarchically porous TiO_2_-supported Au nanoparticles with enhanced visible-light-responsive properties for CO_2_ photoreduction (Fig. 10) [136]. The resultant materials possess well-defined hierarchically porous structures, which are highly interconnected with one another by small pore windows, and the Au nanoparticles are uniformly dispersed and supported on the inner walls. As a result, the obtained materials exhibit high catalytic activity for the photocatalytic reduction of CO_2_ to CH_4_ under visible illumination with a high production rate of 1.48 μmol g^−1^ h^−1^.

### Photoelectrochemical water splitting

**Figure 11.**
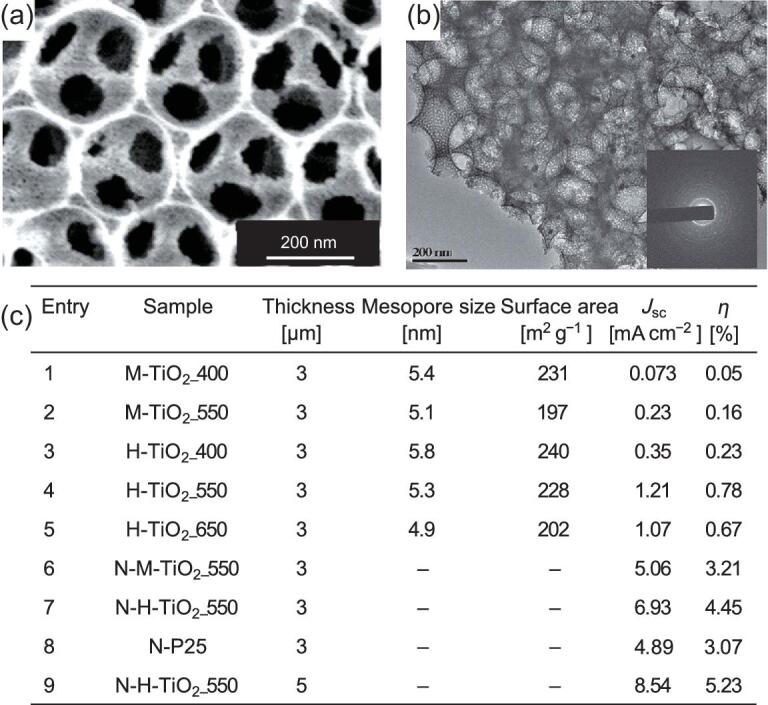
SEM (a) and TEM (b) images of ordered hierarchically macro/mesoporous TiO_2_ films. (c) Photoelectrocatalytic water-splitting performance of the ordered hierarchically macro/mesoporous TiO_2_ films (H-TiO_2_) and the pristine ordered mesoporous TiO_2_ films (M-TiO_2_). Adapted from [71] with permission from Wiley-VCH. Copyright 2014.

Photoelectrochemical (PEC) water splitting, which integrates solar energy collection and water electrolysis into a single photoelectrode, provides a more effective way of H_2_ production than photocatalysis and electrolysis. Mesoporous TiO_2_ films are among the best candidates as a host matrix for PEC water splitting because of their large surface areas, uniform pore sizes, and structural homogeneity and integrity. Construction of macropores in the mesoporous films to produce the hierarchically porous structure can increase the availability of the internal surface and the accessability of active sites, thus improving the photon to current conversion efficiency. Hierarchically porous anatase films with high crystallinity, a high surface area (240 m^2^ g^−1^) and large pore volume (1.2 cm^3^ g^−1^) have been synthesized for PEC cells, which possess a high photocurrent of 1.07 mA cm^−2^ and photoconversion efficiency of 0.67% [71]. After N-doping, the PEC performance of the N-doped hierarchically porous film is further improved because of the extension of the light response, the photocurrent and photoconversion efficiency can increase to 8.54 mA cm^−2^ and 5.23%, respectively (Fig. 11).

Incorporation of quantum dots (QDs) with hierarchically mesoporous TiO_2_ frameworks can improve the PEC performance, too, because of the high separation efficiency of photoinduced charges and holes at the heterointerfaces. Feng *et al.* have used CdS QDs-sensitized hierarchically mesoporous TiO_2_ films as photoanodes for water splitting [67]. An excellent photocurrent density of 6.03 mA cm^−2^ and a photoconversion efficiency of 3.9% can be obtained. A novel photoanode based on N-doped sub-5 nm graphitic pencil nanodots (N-PNDs)-inserted hierarchically mesoporous TiO_2_ films with a high surface area of 97 m^2^ g^−1^, and large pore size of 13.5 nm, have been demonstrated recently [68]. The resultant material exhibits a high photocurrent density of 1.73 mA cm^–2^, a 183% and 108% increase of the values for the pristine and undoped PND-TiO_2_, respectively. Moreover, the incident photo-to-current conversion efficiency (IPCE) measurements show that the N-PND-inserted hierarchically mesoporous TiO_2_ films have an IPCE value of ∼50% over the wavelength range of 325–425 nm, which is more than twice that of the pristine hierarchically mesoporous TiO_2_ films.

### Catalyst support

TiO_2_ is not only the most studied photocatalyst but also a good catalyst support for active metal nanoparticles in many reactions. The thermal stability, high surface areas, and large pore sizes and volumes of hierarchically mesoporous TiO_2_ materials make them ideal catalytic supports. The high surface area can provide more surface active sites, thus improving the activities of catalysts because the chemical reactions take place only when active sites are available on the surface. Besides, the large pore size and volume can facilitate the diffusion of chemicals, promoting the reaction kinetics [137,138].

Hao *et al.* have reported that the hierarchically mesoporous Au/TiO_2_ hybrid nanofibers can efficiently catalyze the reduction of 4-nitrophenol to 4-aminophenol by sodium borohydride [139]. The results show that, with the increase in Au loading, the apparent reaction rate constant is raised but the turnover frequency (TOF) value of the catalyst decreases. This can be attributed to the smaller sizes of Au nanoparticles, which possess higher catalytic activity. By regulation of the architecture and porous structures of mesoporous TiO_2_ materials, the catalytic performance can be improved significantly. For instance, Zhao and coworkers have demonstrated the 3D hierarchically mesoporous bouquet-posy-like TiO_2_/Au as a catalyst for the semihydrogenation of alkynes by using biorenewable formic acid as the hydrogen source [114]. The produced catalyst (0.2 mol% Au) displays 100% diphenylacetylene conversion within 40 min, and the selectivity to *cis*-stilbene is measured to be ≥99%. Moreover, the superstructure catalyst can catalyze a wide range of aromatic and aliphatic terminal alkynes to convert to the corresponding alkenes with a high conversion (≥99.7%) and selectivity (≥96%). Furthermore, the superstructure catalyst can be reused 25 times without performance degradation. The high performance of the hierarchically mesoporous TiO_2_ superstructure can be attributed to the unique structure with more nanoreactor units.

By fabrication of active-nanoparticles@ hierarchically mesoporous TiO_2_ materials the leaching and aggregation of nanoparticles can be prevented in harsh catalytic environments. Lee *et al.* have explored Au@TiO_2_ yolk–shell structures as catalysts for promoting CO oxidation [140]. In this case, the hierarchically porous TiO_2_ shells can act as a physical barrier to prevent the thermal migration and sintering of Au nanoparticles at high temperature. In fact, the Au@TiO_2_ yolk–shell catalyst remains stable upon calcination at high temperatures up to 775 K, with no change in the size and shape of the Au nanoparticles and of the structural integrity of the TiO_2_ shell. In contrast, under similar heat treatment, the reference Au/TiO_2_-P25 catalyst (gold nanoparticles supported on commercial P25-TiO_2_) results in significant sintering and formation of large Au nanoparticles with diameters up to 50 nm. The catalytic results showed that the Au@TiO_2_ catalyst is indeed quite active in promoting the oxidation of carbon monoxide, displaying comparable reaction rates, relative to the exposed surface of the gold nanoparticles, to those obtained with the traditional Au/TiO_2_-P25 catalyst.

### Lithium-ion batteries

Lithium-ion batteries (LIBs) are currently the predominant power source for portable electronics and are expected to be applied in electric vehicles in the near future because of their advantages of high energy density, long lifespan, no memory effect and environmental benignity. TiO_2_ has been considered as a potential alternative material to the traditional graphitic carbon anode because it exhibits excellent Li-ion insertion/extraction reversibility with a low volume change (∼4%) and a high operating voltage ranging from 3 to 1 V (vs. Li/Li^+^), avoiding the formation of solid electrolyte interphase (SEI) layers and the problem of lithium dendrites, thereby yielding better battery safety [141,142]. However, practical applications of TiO_2_ materials for LIBs still present a great challenge due to their low ionic and electrical conductivity.

Employing various nanostructured mesoporous TiO_2_ materials, especially for those with hierarchically porous structures as anodes for rechargeable LIBs, is popular [143]. For instance, hierarchically macro/mesoporous TiO_2_ microparticles possess an excellent initial capacity of 235 and 202 mAh g^−1^ at 0.2 and 1 C, respectively [61]. The reversibility study demonstrates that the hierarchically macro/mesoporous TiO_2_ microparticles display excellent cycling capacity, superior rate behavior and higher coulombic efficiency because the higher surface area provides more active sites and the presence of the inner-particle mesopores serve as a bicontinuous transport path and affords a shorter path length for diffusion of Li ions. The reversible capacity of 106 mAh g^−1^ for the hierarchically macro/mesoporous TiO_2_ microparticles can be retained after 200 charge–discharge cycles at a relatively high current rate of 4 C.

**Figure 12.**
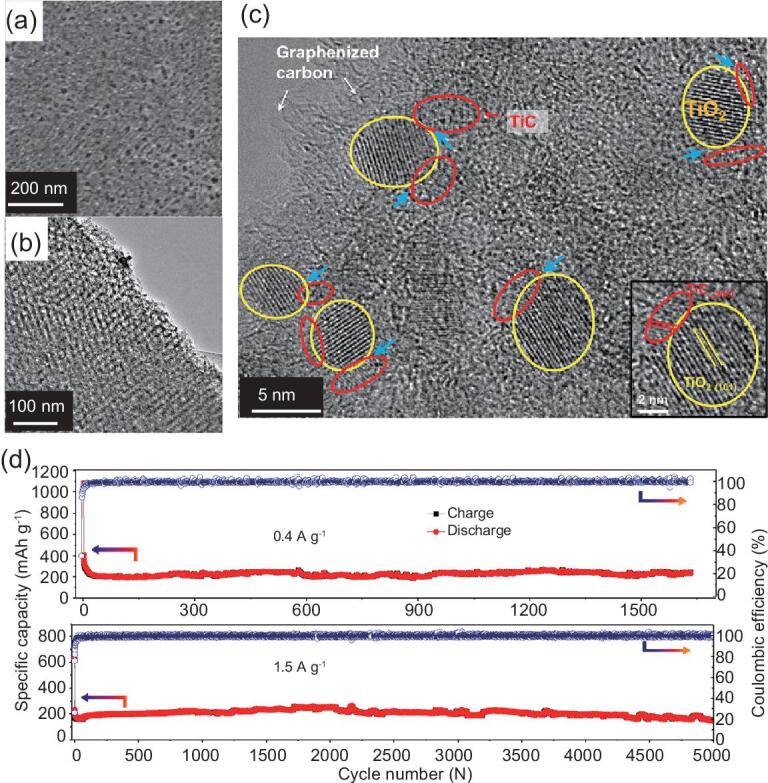
SEM (a), TEM (b) and HRTEM (c) images of hierarchically mesoporous TiO_2_/TiC@C composite synthesized by a facile *in situ* carbothermal reduction method. (d) Cycling performance of the hierarchically mesoporous TiO_2_/TiC@C composite membrane electrodes at current densities of 0.4 and 1.5 A g^−1^ when used as anode for LIBs. Adapted from [144] with permission from Elsevier. Copyright 2018.

To improve the ionic and electrical conductivity of TiO_2_, highly conductive carbon materials, including mesoporous carbon, carbon nanotubes and graphene, are used to combine with TiO_2_ anodes [144,145]. Li *et al.* have reported the preparation of uniform hierarchically mesoporous TiO_2_/graphene/TiO_2_ sandwich-like nanosheets and used them as the anode of LIBs [35]. Because of the small particle size of the primary TiO_2_, the 3D interconnected mesoporosity, the relatively thin layer and the high surface area, the obtained nanosheets deliver an extra high capacity, an excellent high-rate capability and a long cycle life. More recently, hierarchically mesoporous TiO_2_–graphitic carbon nanocomposites have been developed for anode materials [96]. In this case, all TiO_2_ nanocrystals in the synthesized material are conformably encapsulated in ultrathin graphitic carbon layers. Due to the high specific surface area of 298 m^2^ g^−1^, a high pore volume of 0.31 cm^3^ g^−1^, a large pore size of 5 nm and a well-defined hollow structure, the resultant materials achieve excellent electrochemical reactivity and stability. A high specific capacity of 137 mAh g^−1^ can be achieved up to 1000 cycles at a current density of 1 A g^−1^ (5 C). To further improve the electrochemical stability of C–TiO_2_ anodes, for the first time, we have prepared hierarchically mesoporous TiO_2_/TiC@C composites (Fig. 12) [144]. TiC nanodots with high conductivity and electrochemical inactivity at the TiO_2_–C interface can significantly enhance the electrical conductivity and structural stability of the C–TiO_2_ composites. Hence, the TiO_2_/TiC@C membranes deliver a high capacity of 237 mAh g^−1^ at a current density of 0.4 A g^−1^ and an ultra-long cycling life (up to 5000 cycles with over 68.4% reversible capacity retention).

Conducting polymer–TiO_2_ nanocomposites also show great potential for anode materials. Hierarchically mesoporous polyaniline/TiO_2_ spheres with core–shell structure show excellent performance. The discharge capacity of 123.9 and 157.1 mAh g^−1^ can be obtained at the high current density of 1500 and 2000 mA g^−1^, respectively. In addition, some recent reports have shown that the introduction of Ti^3+^ can efficiently improve the intrinsic conductivity of TiO_2_ frameworks [99]. Hierarchically mesoporous Ti^3+^ doped TiO_2_ hollow spheres have demonstrated excellent lithium storage performance with stable capacity retention for over 300 cycles and enhanced rate capability even up to 10 C, which is better than that of the undoped form.

### Sodium-ion batteries

Recently, sodium-ion batteries (SIBs) have drawn great attention and would become one of the low-cost alternatives to LIBs since sodium is earth-abundant and environmental friendly. Owing to their superior safety, stability and sodium storage capability, TiO_2_-based materials have been extensively investigated as anodes [146–149]. For example, hierarchically mesoporous TiO_2_ nanosheets have achieved an excellent reversible capacity of 220 mAh g^−1^ at 100 mA g^−1^. Moreover, the capacity is retained at 44 mAh g^−1^ even at a high current density of 10 A g^−1^ after 10 000 cycles [41].

**Figure 13.**
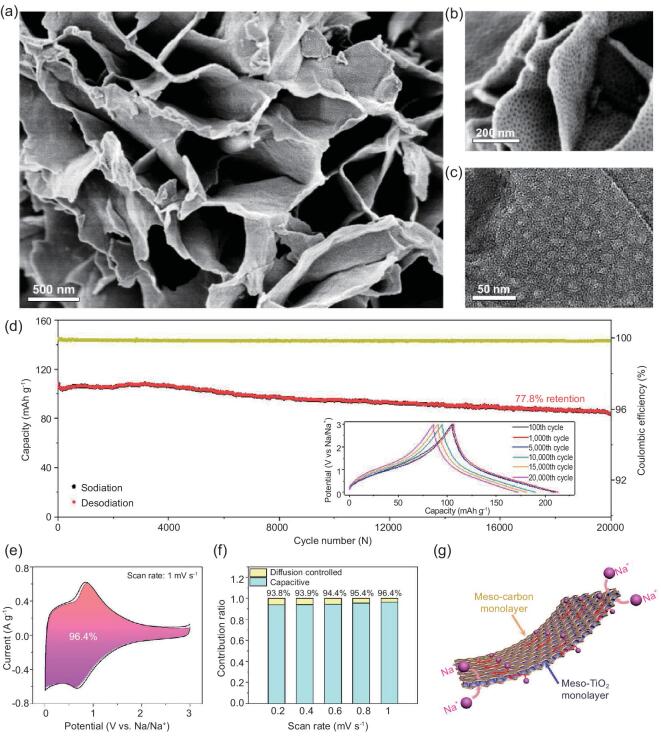
SEM (a, b) and TEM (c) images of hierarchically mesoporous C–TiO_2_ heterostructure. (d) Cycling stability of the hierarchically mesoporous C–TiO_2_ heterostructure after 20 000 charge–discharge cycles at a current density of 10 A g^–1^. (e) Separation of the capacitive and diffusion currents in the hierarchically mesoporous C–TiO_2_ heterostructure at a scan rate of 1 mV s^–1^. (f) Contribution ratio of the capacitive and diffusion-controlled charge vs. scan rates. (g) Schematic model of the hierarchically mesoporous C–TiO_2_ heterostructure, showing charge storage and charge transfer mechanism. Adapted from [151] with permission from the American Chemical Society. Copyright 2019.

To further enhance the performance of TiO_2_ materials for SIBs, it is necessary to increase their conductivity. One effective way is combination with high-conductive carbon [150]. For example, the as-obtained rod-in-tube mesoporous TiO_2_/C nanocomposites with a uniform carbon coating have demonstrated a high discharge capacity of 277.5 and 153.9 mAh g^−1^ at 50 and 5000 mA g^−1^, respectively, and almost 100% capacity retention over 14 000 cycles at 5000 mA g^−1^. More recently, Zhao and coworkers have reported that the rate capability and cyclability of mesoporous TiO_2_ for SIBs can be further improved by producing mesopores in the coated carbon layers to produce the hierarchically mesoporous C–TiO_2_ heterostructure (Fig. 13) [151]. The hierarchically mesoporous vertical heterostructure, which consists of well-ordered monolayered mesoporous TiO_2_ nanosheets surrounded by two mesoporous carbon monolayers, provides a highly accessible surface area for effective access of electrolyte and well-defined interstices for volume strain, enabling excellent rate capability and cyclability. Moreover, a pseudocapacitive contribution of 96.4% at a low sweep rate of 1 mV s^–1^ can be achieved because of the efficient electrochemical faradaic redox resulting from the efficient interfacial electron transfer.

## CONCLUSION AND PERSPECTIVES

Undoubtedly, hierarchically mesoporous TiO_2_ materials with various pore structures and morphologies show great potential in many fields, which can get more attention in the future. In this review, we summarized the recent advances in the controllable synthesis and applications of hierarchically mesoporous TiO_2_ materials. The synthetic routes and formation mechanisms of hierarchically mesoporous TiO_2_ materials with different architectures are summarized first. Then, the applications of hierarchically mesoporous TiO_2_ materials in energy and environmentally related areas, such as photocatalytic degradation of pollutants, photocatalytic fuel generation, photoelectrochemical water splitting, catalyst support, lithium-ion batteries and sodium-ion batteries, are discussed in detail based on the structure–performance relationship.

However, some key scientific problems still remain in the preparation of hierarchically mesoporous TiO_2_ materials. Firstly, the synthetic processes and mechanisms for hierarchically mesoporous TiO_2_ materials require further in-depth understanding at the atomic level, laying a solid foundation to achieve the precise synthesis of hierarchically mesoporous TiO_2_ materials with desired structures at nanoscale, even sub-nanoscale. Therefore, developing techniques to monitor the real-time and real-space growing process of hierarchically mesoporous TiO_2_ materials in solution is essential. Secondly, a facile and reliable approach for the synthesis of hierarchically mesoporous TiO_2_ materials with well-controlled mesostructures, pore sizes and architectures is still in demand. A promising breakthrough is based on the super-assembly of unconventional building blocks, such as monomicelles, nanoparticles and nanoclusters. Those building blocks can act as ‘artificial atoms’ and then hierarchically mesoporous materials with complex mesostructures, like *Ia3d*, or morphologies, like Janus, satellite-like core-shell can be fabricated. Thirdly, the crystal phase control of hierarchically mesoporous TiO_2_ materials should gain more attention. Based on the theoretical prediction, there are more than 20 crystal phases of TiO_2_. Tuning the cross-link manner of the [TiO_6_] octahedron during the cooperative assembly process can produce hierarchically mesoporous TiO_2_ materials with new crystal phases and then change their physical and chemical properties significantly.

Concerning applications, the central task is to develop a reliable structure–performance relationship that can guide the synthesis and rational design of hierarchically mesoporous TiO_2_ materials. In the photoconversion area, which is the most promising for the practical application of TiO_2_ materials, there is still a demand for further increasing the utilization of sunlight. To this end, it is necessary to the fabrication of highly efficient heterojunctions with more complex structures such as tandem junctions with perfect band alignment and/or full-spectrum absorption. In addition, the coupling of light and electricity/heat in an integrated system is a promising method. In terms of batteries, the low conductivity and capacity of the TiO_2_ materials severely limit their performances. Hybridization of TiO_2_ materials with other components that possess a high conductivity and capacity to achieve a long cycle life, excellent rate performance and high energy density are necessary. Besides, the high surface area and porous structure of the hierarchically mesoporous material can result in side reactions and low volume energy density. Therefore, the fabrication of advanced nanostructures of hierarchically mesoporous TiO_2_ materials to balance the porous structure and volume energy density is very important. Furthermore, the large pore volumes make the hierarchically mesoporous TiO_2_ materials as ideal hosts for active materials, using hierarchically mesoporous TiO_2_ materials to develop novel battery systems, such as lithium–sulfur, lithium metal and sodium metal batteries, is a potential field. For catalyst support, the essential issue is the fabrication of hierarchical porous TiO_2_ materials with ultrahigh surface areas (>300 m^2^ g^−1^), which can facilitate the diffusion of reactants and products, thus improving the catalytic efficiency.

Overall, the development of hierarchically mesoporous TiO_2_ materials provides new opportunities for overcoming the energy and environmental issues in our lives. We hope that, in the future, significant breakthroughs can be made in research areas from synthetic, fundamental and practical viewpoints.
